# Implications of a high-definition multileaf collimator (HD-MLC) on treatment planning techniques for stereotactic body radiation therapy (SBRT): a planning study

**DOI:** 10.1186/1748-717X-4-22

**Published:** 2009-07-10

**Authors:** James A Tanyi, Paige A Summers, Charles L McCracken, Yiyi Chen, Li-Chung Ku, Martin Fuss

**Affiliations:** 1 Department of Radiation Medicine, Oregon Health & Science University, Portland, OR 97239, USA; 2 Department of Nuclear Engineering & Radiation Health Physics, Oregon State University, Corvallis, OR 97331, USA; 3 Department of Physics, Santa Clara University, Santa Clara, CA 95053, USA; 4 Department of Public Health & Preventive Medicine, Oregon Health & Science University, Portland, OR 97239, USA

## Abstract

**Purpose:**

To assess the impact of two multileaf collimator (MLC) systems (2.5 and 5 mm leaf widths) on three-dimensional conformal radiotherapy, intensity-modulated radiotherapy, and dynamic conformal arc techniques for stereotactic body radiation therapy (SBRT) of liver and lung lesions.

**Methods:**

Twenty-nine SBRT plans of primary liver (n = 11) and lung (n = 18) tumors were the basis of this study. Five-millimeter leaf width 120-leaf Varian Millennium (M120) MLC-based plans served as reference, and were designed using static conformal beams (3DCRT), sliding-window intensity-modulated beams (IMRT), or dynamic conformal arcs (DCA). Reference plans were either re-optimized or recomputed, with identical planning parameters, for a 2.5-mm width 120-leaf BrainLAB/Varian high-definition (HD120) MLC system. Dose computation was based on the anisotropic analytical algorithm (AAA, Varian Medical Systems) with tissue heterogeneity taken into account. Each plan was normalized such that 100% of the prescription dose covered 95% of the planning target volume (PTV). Isodose distributions and dose-volume histograms (DVHs) were computed and plans were evaluated with respect to target coverage criteria, normal tissue sparing criteria, as well as treatment efficiency.

**Results:**

Dosimetric differences achieved using M120 and the HD120 MLC planning were generally small. Dose conformality improved in 51.7%, 62.1% and 55.2% of the IMRT, 3DCRT and DCA cases, respectively, with use of the HD120 MLC system. Dose heterogeneity increased in 75.9%, 51.7%, and 55.2% of the IMRT, 3DCRT and DCA cases, respectively, with use of the HD120 MLC system. DVH curves demonstrated a decreased volume of normal tissue irradiated to the lower (90%, 50% and 25%) isodose levels with the HD120 MLC.

**Conclusion:**

Data derived from the present comparative assessment suggest dosimetric merit of the high definition MLC system over the millennium MLC system. However, the clinical significance of these results warrants further investigation in order to determine whether the observed dosimetric advantages translate into outcome improvements.

## Background

Stereotactic body radiation therapy (SBRT) is a modern precision radiation therapy delivery concept characterized by one to five fraction delivery of focal high-dose radiation [1,2]. SBRT has become an established treatment technique for lung [3-5], liver [6-8], and spinal lesions [9-11]. Conceptually derived from cranial stereotactic radiosurgery, the planning and delivery of SBRT is characterized by highly target-conformal dose distributions with steep dose gradients towards normal tissues, which allow the administration of potent tumor-ablative radiation doses.

Beam shaping for linear accelerator-based SBRT planning and delivery is mostly afforded by multileaf collimator (MLC) systems. Over the last 15 years, MLCs have evolved in terms of both field size and width of the individual tungsten leafs, and it is intuitive to assume that target dose conformity and/or the steepness of the dose gradient can be influenced by decreasing MLC leaf width [12-23]. The current work seeks to assess if a novel high-definition 2.5-mm leaf MLC system (HD-MLC) integrated into a dedicated stereotactic linear accelerator system (BrainLAB/Varian Novalis TX) provides dosimetric advantages compared with a clinically widely utilized 5 mm leaf system for SBRT of lung and liver lesions, and if potential gains realized may be clinically meaningful.

## Materials and methods

### Patients and treatment protocol

The present study is based on 29 patients that had undergone a course of SBRT at Oregon Health & Science University in Portland, Oregon, USA between July 2007 and May 2008. The patient population included 18 primary early stage lung tumors and 11 hepatocellular carcinoma (HCC). Clinical treatment planning simulation imaging and SBRT delivery were performed with patients immobilized in a double vacuum whole-body immobilization system (BodyFix; Medical Intelligence, Schwabmuenchen, Germany). The basis for SBRT was thin slice CT scans acquired on a dedicated 16 slice big-bore CT simulator (Philips Medical Systems, Cleveland, OH, USA). The imaging data was electronically transferred to the Eclipse radiation therapy planning system (Varian Medical Systems, Palo Alto, CA, USA). Based on both free-breathing and respiration resolved 4DCT scans, the internal target volume (ITV) was delineated and expanded into a planning target volume (PTV) by adding isotropic 5 mm margins. All clinical SBRT plans (reference plans) were computed using a multiple static field sliding-window IMRT technique for delivery on the Varian Trilogy platform (Varian Medical Systems, Palo Alto, CA) equipped with a 120-leaf Millennium MLC (M120 MLC) system, with forty 5-mm central leaf-pairs and twenty 10-mm peripheral leaf-pairs. The anisotropic analytical algorithm (AAA) was used for dose computation with a dose calculation grid of 2.5 mm^3^. Tissue heterogeneity was taken into account. All treatments were planned for five fraction delivery (10 Gy/fraction for liver tumors, and 12 Gy/fraction for lung lesions). All plans were computed such that the prescribed dose (PD) encompassed 95% of the PTV, with a heterogeneous dose distribution and a desired plan maximum of 150% of PD.

Comparative plans were generated from corresponding reference IMRT plans by re-optimization for the Novalis TX treatment platform (Varian Medical Systems), equipped with a high-definition MLC (HD120 MLC) system with thirty-two 2.5-mm central leaf-pairs and twenty-eight 5-mm peripheral leaf-pairs. To assure valid data generation, all reference plans were carefully selected from a larger library of SBRT plans to ensure that PTVs were conformed by the central 5 mm leafs of the Varian Trilogy platform, and correspondingly, only the central 2.5 mm leafs of the Novalis TX platform for the comparative plans.

In addition to the influence of the respective MLC system on IMRT-based SBRT dose distributions, the impact of MLC system was also investigated for commonly utilized static three-dimensional conformal radiation therapy (3DCRT), and dynamic conformal arc (DCA) planning techniques. Hence, besides the available M120 MLC IMRT reference plan, the following five alternative treatment plans were generated for each patient: (1) HD120 MLC IMRT, (2) M120 MLC 3DCRT, (3) HD120 MLC 3DCRT, (4) M120 MLC DCA, and (5) HD120 MLC DCA. Nine to twelve beams were used to generate the IMRT and 3DCRT plans. Beam angles were arranged in a practical manner according to tumor and critical organ location for the purpose of achieving maximal target coverage and optimal dose distribution conformity while keeping doses to OAR (including the contralateral lung, liver, spinal cord, esophagus, bowel, and ipsilateral kidney) below institutional dose limits.

### Evaluation parameters

All study cases were categorized into five groups according to ITVs: category O; all ITVs, category I; 1 ≤ ITV < 8 cm^3^, category II; 8 ≤ ITV < 27 cm^3^, category III; 27 ≤ ITV < 64 cm^3^, and category IV; ITV ≥ 64 cm^3^. Categories I though IV were selected because they each equaled the volumes of cubes with side length of 1, 2, 3, and 4 cm, respectively [19].

Each treatment plan was evaluated with respect to target coverage criteria, normal tissue sparing criteria, as well as treatment efficiency. In terms of target coverage criteria, PTV dose-volume histogram (DVH) parameters including mean dose (or D_mean_, defined in this study as the sum of the product of dose value and percent volume in each dose bin), minimum dose (or D_min_, defined in this study as dose to 99% of the PTV) and maximum dose (or D_max_, defined in this study as dose received by the "hottest" 3% volume of the PTV) were computed and recorded. The conformity of each treatment plan was quantified using a robust conformity index (CI) based on formulations by Paddick [24] and Nakamura *et al*. [25]

(1)

where PIS is the prescription isodose surface, V_PTV _is the magnitude of the planning target volume, V_PIS _is the volume encompassed by the prescription isodose surface, and PTV_PIS _is the planning target volume encompassed within the prescription isodose surface. Since all plans in the current study were normalized such that 95% of the planning target volume was conformally covered by the prescription isodose surface, the PTV_PIS _is 95% of the V_PTV_. Also, target dose heterogeneity was assessed using a heterogeneity index (HI) define below:

(2)

By considering normal tissue outside the PTV but in the dose volume space as a virtual structure, *dose-spillage volumes *[26] were computed to assess normal tissue sparing effect of the MLC systems. The following dose spillage volumes were assessed: 1) V_HS _or high-dose spillage volume taking into account normal tissue receiving an ablative dose; that is, ≥ 90% of the prescription dose in the current study, 2) V_IS _or intermediate-dose spillage volume taking into account normal tissue receiving a significant fraction of the prescription dose; that is, ≥ 50% of the prescription dose, and 3) V_LS _or low-dose spillage volume taking into account normal tissue receiving low doses of radiation; that is, ≥ 25% of the prescription dose.

Finally, the efficiency of each treatment plan was computed as a ratio of the cumulative sum of monitor units (MUs) per fraction to the dose per fraction. A paired *t*-test with two-tailed distribution, and a *p*-value < 0.05 defining statistical significance, was used to assess whether differences between the MLC systems were statistically significant.

## Results

### Target dose-volume parameters

**Figure 1:**
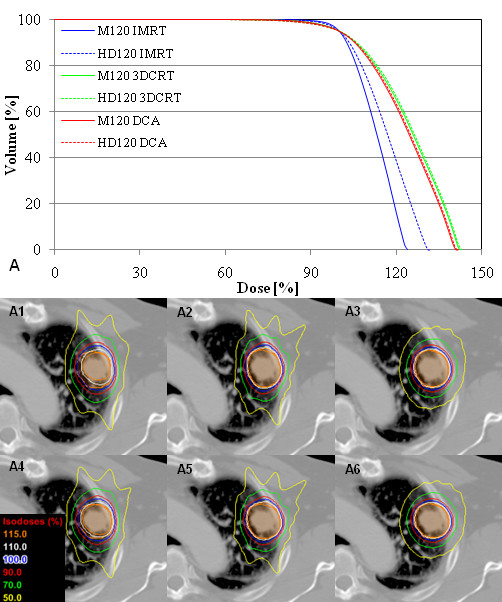
**Isodose distributions and DVHs for a lung lesion generated from three different planning techniques and two MLC systems**. A1 through A6 are axial isodose distribution corresponding to M120 MLC IMRT, M120 MLC 3DCRT, M120 MLC DCA, HD120 MLC IMRT, HD120 MLC 3DCRT, and HD120 MLC DCA plans, respectively.

The median ITV and PTV for all 29 cases in the current study were 7.58 cm^3 ^[range: 1.03–91.53 cm^3^] and 26.33 cm^3 ^[range: 13.95–167.44 cm^3^], respectively. The DVHs and corresponding isodose distributions for all involved treatment planning techniques are shown for a representative lung cancer case in Figure 1. Additional file 1 summarizes the median mean, minimal and maximal PTV doses for each planning technique, separated in terms of treatment site and MLC system. Overall, there was demonstrable quantitative difference between corresponding HD120 MLC and M120 MLC PTV doses, although not every perceived difference was statistically significant.

### Target dose conformity and normal/critical structure dose

The mean values of the conformity and heterogeneity indices, along with *p*-values of paired *t*-tests comparing corresponding planning techniques of the MLC systems under consideration, are summarized in Additional file 2 according to ITV groups. Overall, HD120 MLC plans exhibited better conformity than M120 MLC plans. Unlike the IMRT cases where no clear trend was exhibited for the mean conformity and heterogeneity indices, plans of both 3DCRT and DCA showed a decreasing pattern with increasing ITV. Furthermore, the conformity index either stayed the same or increased with increasing MLC leaf width. However, unlike the conformity index, the heterogeneity index either stayed the same or decreased with increasing MLC leaf width. Despite these perceived quantitative differences, all but two the *p*-values of paired t-tests of the conformity index between the different MLC plans were greater than 0.05.

Additional file 3 summarizes the median dose to OAR (including the spinal cord, esophagus, ipsilateral kidney, ipsilateral lung, and liver). For the spinal cord and the esophagus, the magnitude of the range of values was determined by the proximity of the OAR to the PTV. The volume of normal tissue irradiated to ≥ 90%, ≥ 50% and ≥ 25% of the prescription dose, normalized to the planning target volume, is summarized in Table 1, along with *p*-values of paired *t*-tests comparing corresponding planning techniques of the MLC systems under consideration. The results indicate an overall lower dose spillage from the HD120 MLC compared with the M120 MLC. The number and percentage of patient plans with improved performance of the HD120 MLC over the M120 MLC are shown in Table 2, while Table 3 summarizes the mean and maximum absolute percent improvement.

**Table 1:** Mean dose-spillage volume, normalized to PTV.*p*-values of the paired *t*-test included to assess difference between MLC systems.

Technique	V_HS_		V_IS_		V_LS_	
	
	M120	HD120	M120	HD120	M120	HD120
IMRT	0.54 ± 0.30	0.50 ± 0.25	3.86 ± 1.38	3.66 ± 1.22	23.69 ± 9.21	23.14 ± 8.75
	*p *= 0.07		*p *= 0.03		*p *= 0.08	
3DCRT	0.47 ± 0.13	0.44 ± 0.10	4.08 ± 1.34	3.93 ± 1.12	23.64 ± 7.70	23.36 ± 7.70
	p = 0.04		p = 0.24		p = 0.01	
DCA	0.44 ± 0.13	0.43 ± 0.12	3.26 ± 0.61	3.19 ± 0.60	15.32 ± 4.36	14.76 ± 4.23
	*p *= 0.34		*p *= 0.06		*p *= 0.03	

**Table 2:** Cases where performance of HD120 MLC surpassed that of M120 MLC.

Technique	CI	HI	V_HS_	V_IS_	V_LS_
IMRT	15 (51.7%)	22 (75.9%)	19 (65.5%)	24 (82.8%)	22 (75.9%)
3DCRT	18 (62.1%)	15 (51.7%)	21 (72.4%)	21 (72.4%)	25 (86.2%)
DCA	16 (55.2%)	16 (55.2%)	20 (69.0%)	23 (79.3%)	23 (79.3%)

The values in the table are presented as the number of cases and their corresponding ratio (as a percentage) over the 29 patient cases assessed in the current study.

**Table 3:** Mean (top) and max (bottom) percent improvement or worsening of HD120 MLC plans over M120 MLC plans.

Technique	Improvement (%)				Worsening (%)			
	
	CI	V_HS_	V_IS_	V_LS_	CI	V_HS_	V_IS_	V_LS_
IMRT	3.9	4.6	5.5	3.5	2.1	3.1	8.5	5.1
3DCRT	2.5	2.5	4.6	1.8	2.2	2.0	5.8	2.6
DCA	2.4	2.2	3.0	3.3	2.7	2.7	4.0	5.1

IMRT	18.5	20.4	26.5	22.7	10.4	17.7	27.6	14.0
3DCRT	9.5	9.8	39.4	3.7	13.2	8.7	25.9	3.7
DCA	8.1	6.4	9.4	9.6	13.2	9.8	12.1	34.3

### Planning efficiency

The mean value of the total number of MUs necessary to deliver the prescribed dose per fraction for all patients and respective treatment plan category are presented in Table 4.

**Table 4:** Mean number of monitor units (within one standard deviation) necessary to deliver one centigray of prescribed dose for different treatment plan categories.

Technique	IMRT		3DCRT		DCA	
	
	M120	HD120	M120	HD120	M120	HD120
MU/cGy (μ ± σ)	3.45 ± 1.06	3.63 ± 1.36	2.25 ± 0.54	2.26 ± 0.54	2.24 ± 0.54	2.28 ± 0.56
	*p *= 0.17		*p *= 0.80		*P *= 0.18	

The mean MU/cGy for the HD120 MLC system was slightly higher for IMRT plans. However, there was virtually no difference between the MLC systems for the 3DCRT and DCA cases.

## Discussion

One of the most compelling studies to assess the impact of MLCs on dose distributions was performed by Bortfeld *et al*. [15]. The authors show that the theoretically calculated optimal leaf width for a 6 MV photon beam is in the range of 1.5–2 mm. Of all the practical studies that have been conducted, there is utter agreement that by changing MLC widths from 10 mm to 5–3 mm the results are both statistically and clinically significant [12,13,17-21]. Dosimetric improvements reported by such studies, if applied to the SBRT process, may reduce chronic normal/critical structure injuries as the percentage volume of these structures receiving all ranges of dose is in effect reduced. Furthermore, for the PTV, increased maximum dose and improved dose conformity may benefit SBRT as an ablative process. Nevertheless, the quantitation of any advantage obtained by smaller leaf width MLC systems over the 5 mm leaf width MLC has remained controversial [13,14,16,19,20,23].

In the present study, the potential clinical benefit of a novel 2.5 mm leaf width MLC system over a clinically available 5 mm leaf width MLC system was explored for different SBRT treatment planning techniques of lung and liver lesions. A variety of target dose parameters were considered, including mean, minimum and maximum PTV doses; conformity and heterogeneity indices; and normal tissue sparing. Wu *et al*. [23], in a similar study on a subset of five liver cancer patients, showed that the HD120 MLC system has no significant impact on D_min_, D_max_, or D_mean _values relative to the M120 MLC system. These results were in agreement with findings in the current study. Nonetheless, unlike results in Additional file 1 of the current study, Wu *et al*. [23] reported significantly reduced D_max _values for the liver patient subgroup (p < 0.01) with use of IMRT and the HD120 MLC system, albeit small (<2%) compared with the M120 MLC system.

Regarding dose distribution conformity, results in Additional file 2 demonstrated an improvement in conformity index with target volume for all assessed planning techniques. The IMRT technique showed the best PTV coverage of either MLC system, except for large targets (defined in the current study as ITV ≥ 64 cm^3^). As indicated in Tables 2 and 3, in 51.7% of the IMRT cases, use of the HD120 MLC improved the conformality of the original plans by a mean value of 3.9% and up to a maximum value of 18.5%. In 62.1% and 55.2% of the 3DCRT and DCA cases, respectively, use of the HD120 MLC also resulted in improved PTV dose conformality. The mean and maximum improvements were 2.5% and 9.5% for the 3DCRT technique, and 2.4% and of 8.1% for the DCA technique, respectively. Nevertheless, the conformity index difference between the MLC systems is quite small, regardless of the treatment planning technique (see Additional file 2), attributable in part to the number of beams used for treatment planning.

Normal tissue sparing effect of the MLC systems was assessed, by considering normal tissue outside the PTV but in the dose volume space as a virtual structure. Similar to findings by Wu *et al*. [23], a reduction in normal tissue dose was observed with the HD120 MLC system, with at least 19 of the 29 cases per treatment planning technique having lower volumes exposed to the 90%, 50% and 25% dose levels. To be specific, at least 65.5%, 72.4%, and 75.9% cases per planning technique had lower normal tissue volumes exposed to the V_HS_, V_IS_, and V_LS_, respectively (see Table 2). The mean dose reduction attributable to the HD120 MLC was between 1 – 4% for the 3DCRT and DCA techniques, and between 2 – 6% for the IMRT technique. Thus, in terms of dose reduction, the IMRT plans were apparently better than either 3DCRT or DCA plans. However, the quantitative normal tissue volumes exposed to the 90%, 50% and 25% dose levels were smallest for the DCA technique, irrespective of MLC system.

Regarding treatment planning efficiency, while the 3DCRT and DCA techniques showed little difference in treatment monitor units between MLC systems, results in the current study indicated an increase in monitor units, albeit statistically insignificant, with the HD120 MLC system for the IMRT technique. This was attributable to an increase in the number of MLC segments needed to deliver the prescribed dose [12,20].

On a final note, the current work is purely a treatment planning study on a single treatment planning platform with no dosimetric verification. The dosimetric differences reported here are believed to be solely due to the different leaf widths used in the treatment planning, since our comparisons were performed on the same treatment planning system for two treatment platforms with similar open-field beam characteristics, using the same beam configurations, optimization parameters (for IMRT), and dose constraints. Nevertheless, it should be pointed out that leaf-width is not the only parameter that is different between these MLC systems. Factors such as the leaf transmission and leakage (a function of leaf height, material constituent, and tongue-and-groove), source-to-MLC distance, are also different and affect dosimetric parameters. Therefore, the current planning study is not a simple comparison for different MLC leaf-widths, but rather a complex comparison of two dose delivery systems with different leaf-width MLCs [19].

## Conclusion

Data derived from the present comparative assessment suggest dosimetric merit of the high definition MLC system over the millennium MLC system. However, the clinical significance of these results warrants further investigation in order to determine whether the observed dosimetric advantages translate into outcome improvements.

## Competing interests

MF: Varian Medical Systems, Palo Alto, CA; Research support, Consultant, Speaker.

## Authors' contributions

JAT participated in the conception and design of the study, performed data analysis, evaluated the results and drafted the manuscript. PAS was responsible for data acquisition and revised the manuscript. YC participated in the statistical analytical assessment of the data. CLM was responsible for data acquisition and revised the manuscript. LK participated in the design of the study and revised the manuscript. MF treated all the patients that form the basis of this study, participated in the design of the study and data analysis and revised the manuscript. All authors read and approved the final manuscript.

## Supplementary Material

Additional file 1 **Supplementary table**. Median value and range of target dose parameters, expressed as a percent of the prescription dose. Click here for file

Additional file 2 **Supplementary table**. Group-based analyses of mean conformity and heterogeneity indices for each MLC plan. Click here for file

Additional file 3 **Supplementary table**. Median value and range of organ-at-risk (OAR) dose as a percent of the prescription dose. Click here for file
