# Supplementary material for: Implications of a high-definition multileaf collimator (HD-MLC) on treatment planning techniques for stereotactic body radiation therapy (SBRT): a planning study
Source: Radiat Oncol. 2009 Jul 10;4:22. doi: 10.1186/1748-717X-4-22 (PMC2716348; doi:10.1186/1748-717X-4-22)
Supplement: Additional file 1 — Supplementary table. Median value and range of target dose parameters, expressed as a percent of the prescription dose. [file 1748-717X-4-22-S1.doc]

Additional file 1: Median value and range of target dose parameters, expressed as a percent of the prescription dose. *p*-values of the paired *t*-test included to assess the difference between corresponding M120 and HD120 MLC plans.

| Site | Technique | D_mean_ (%) | | D_min_ (%) | | D_max_ (%) | |  | |
| --- | --- | --- | --- | --- | --- | --- | --- | --- | --- |
|  |  | M120 | HD120 | M120 | HD120 | M120 | HD120 |  | |
| Lung  (n=18) | IMRT | 118.9  [110.8 – 132.5] | 119.0  [111.7 – 133.0] | 94.9  [75.4 – 98.7] | 94.0  [72.6 – 98.6] | 147.1  [122.8 – 169.1] | 147.0  [125.6 – 170.4] |  | |
|  |  | *p* = 0.02 | | *p =* 0.02 | | *p* = 0.02 | | *p* = 0.02 | *p =* 0.02 |
|  | 3DCRT | 128.5  [120.4 – 136.7] | 127.8  [118.2 – 134.8] | 86.7  [71.7 – 94.7] | 85.9  [75.8 – 90.6] | 149.5  [133.5 – 164.6] | 147.7  [130.4 – 161.8] |  | |
|  |  | *p* = 0.05 | | *p =* 0.51 | | *p* = 0.05 | | *p* = 0.05 | *p =* 0.51 |
|  | DCA | 126.9  [120.3 – 139.9] | 127.3  [118.4 – 136.9] | 83.8  [73.6 – 94.8] | 82.7  [59.9 – 87.0] | 147.5  [130.3 – 170.6] | 146.7  [127.3 – 165.8] |  | |
|  |  | *p =* 0.53 | | *p =* 0.05 | | *p =* 0.53 | | *p =* 0.53 | *p =* 0.05 |
| Liver  (n=11) | IMRT | 114.7  [109.6 – 132.5] | 113.7  [109.7 – 133.0] | 96.7  [93.6 – 100.9] | 96.5  [92.9 – 100.3] | 132.9  [114.1 – 170.9] | 132.9  [113.7 – 170.4] |  | |
|  |  | *p =* 0.31 | | *p =* 0.02 | | *p =* 0.31 | | *p =* 0.31 | *p =* 0.02 |
|  | 3DCRT | 125.3  [118.3 – 136.7] | 124.9  [119.8 – 136.3] | 90.4  [76.9 – 132.4] | 83.1  [79.8 – 93.5] | 140.5  [128.7 – 164.6] | 139.3  [130.4 – 161.8] |  | |
|  |  | *p =* 0.77 | | *p =* 0.07 | | *p =* 0.77 | | *p =* 0.77 | *p =* 0.07 |
|  | DCA | 124.0  [111.5 – 133.0] | 123.2  [112.5 – 136.6] | 83.9  [64.2 – 93.0] | 81.5  [72.3 – 92.9] | 137.4  [117.9 – 148.5] | 134.0  [119.1 – 152.2] |  | |
|  |  | *p =* 0.36 | | *p =* 0.83 | | *p =* 0.18 | |  | |
