# Supplementary material for: Implications of a high-definition multileaf collimator (HD-MLC) on treatment planning techniques for stereotactic body radiation therapy (SBRT): a planning study
Source: Radiat Oncol. 2009 Jul 10;4:22. doi: 10.1186/1748-717X-4-22 (PMC2716348; doi:10.1186/1748-717X-4-22)
Supplement: Additional file 2 — Supplementary table. Group-based analyses of mean conformity and heterogeneity indices for each MLC plan. [file 1748-717X-4-22-S2.doc]

Additional file 2: Group-based analyses of mean conformity and heterogeneity indices for each MLC plan. *p*-values of the paired *t*-test included to assess the difference between corresponding M120 and HD120 MLC plans.

| Category | Conformity Index | | | | | | Heterogeneity Index | | | | | |
| --- | --- | --- | --- | --- | --- | --- | --- | --- | --- | --- | --- | --- |
|  | IMRT | | 3DCRT | | DCA | | IMRT | | 3DCRT | | DCA | |
|  | M120 | HD120 | M120 | HD120 | M120 | HD120 | M120 | HD120 | M120 | HD120 | M120 | HD120 |
| O | 1.27±0.19 | 1.25±0.16 | 1.32±0.11 | 1.31±0.08 | 1.29±0.14 | 1.29±0.13 | 0.38±0.12 | 0.39±0.13 | 0.46±0.08 | 0.47±0.06 | 0.47±0.09 | 0.49±0.08 |
| (n=29) | *p* = 0.22 | | *p* = 0.23 | | *p* = 0.72 | | *p* = 0.02* | | *p* = 0.37 | | *p* = 0.16 | |
| I | 1.29±0.22 | 1.28±0.18 | 1.35±0.12 | 1.33±0.08 | 1.34±0.11 | 1.34±0.08 | 0.37±0.11 | 0.38±0.11 | 0.49±0.08 | 0.49±0.06 | 0.51±0.07 | 0.52±0.08 |
| (n=16) | *p* = 0.67 | | *p* = 0.39 | | *p* = 0.77 | | *p* = 0.04** | | *p* = 0.77 | | *p* = 0.17 | |
| II | 1.16±0.06 | 1.14±0.07 | 1.34±0.10 | 1.32±0.10 | 1.30±0.10 | 1.30±0.09 | 0.39±0.16 | 0.40±0.16 | 0.45±0.08 | 0.47±0.04 | 0.49±0.06 | 0.49±0.05 |
| (n=6) | *p* = 0.51 | | *p* = 0.15 | | *p* = 0.81 | | *p* = 0.14 | | *p* = 0.31 | | *p* = 0.95 | |
| III | 1.23±0.08 | 1.23±0.09 | 1.26±0.03 | 1.26±0.04 | 1.27±0.09 | 1.25±0.07 | 0.43±0.19 | 0.46±0.13 | 0.42±0.07 | 0.42±0.03 | 0.41±0.05 | 0.42±0.01 |
| (n=4) | *p* = 0.61 | | *p* = 0.96 | | *p* = 0.64 | | *p* = 0.22 | | *p* = 0.98 | | *p* = 0.77 | |
| IV | 1.42±0.19 | 1.31±0.12 | 1.23±0.08 | 1.24±0.07 | 1.03±0.22 | 1.03±0.22 | 0.39±0.17 | 0.38±0.16 | 0.37±0.02 | 0.41±0.05 | 0.33±0.04 | 0.38±0.04 |
| (n=3) | *p* = 0.35 | | *p* = 0.80 | | *p* = 0.99 | | *p* = 0.57 | | *p* = 0.34 | | *p* = 0.39 | |

*The difference (HD120 minus M120) of the means is 0.012. ** The difference (HD120 minus M120) of the means is 0.010.
