# Supplementary material for: Implications of a high-definition multileaf collimator (HD-MLC) on treatment planning techniques for stereotactic body radiation therapy (SBRT): a planning study
Source: Radiat Oncol. 2009 Jul 10;4:22. doi: 10.1186/1748-717X-4-22 (PMC2716348; doi:10.1186/1748-717X-4-22)
Supplement: Additional file 3 — Supplementary table. Median value and range of organ-at-risk (OAR) dose as a percent of the prescription dose. [file 1748-717X-4-22-S3.doc]

Additional file 3: Median value and range of organ-at-risk (OAR) dose as a percent of the prescription dose. *p*-values of the paired *t*-test included to assess the difference between corresponding M120 and HD120 MLC plans. *D*_max_ = maximum dose to the spinal cord, *D*_5cc_ = dose to 5 cm^3^ of the esophagus, *D*_33%_ = dose to 33% of the ipsilateral kidney, *D*_50%_ = dose to 50% of the ipsilateral lung, and *D*_700cc_ = dose to 700 cm^3^ of the liver.

| OAR | IMRT | | 3DCRT | | DCA | |
| --- | --- | --- | --- | --- | --- | --- |
|  | M120 | HD120 | M120 | HD120 | M120 | HD120 |
| Spinal Cord (*D*_max_)  (n = 29) | 32.1  [1.5 – 59.3] | 31.9  [1.4 – 53.6] | 33.9  [0.9 – 67.1] | 37.4  [1.0 – 62.6] | 27.9  [4.0 – 60.1] | 28.0  [4.3 – 58.2] |
|  | *p* = 0.66 | | *p* = 0.42 | | *p* = 0.06 | |
| Esophagus (*D*_5cc_)  (n = 16) | 16.1  [2.3 – 57.5] | 16.2  [1.2 – 50.1] | 13.3  [1.5 – 67.4] | 12.8  [1.5 – 65.7] | 21.7  [5.9 – 60.9] | 20.7  [5.9 – 59.2] |
|  | *p* = 0.12 | | *p* = 0.02 | | *p* = 0.16 | |
| Ipsilateral Kidney (*D*_33%_)  (n = 9) | 0.8  [0.5 – 2.5] | 0.8  [0.4 – 2.5] | 0.7  [0.4 – 2.5] | 0.7  [0.4 – 2.3] | 0.7  [0.4 – 9.3] | 0.6  [0.4 – 9.3] |
|  | *p* = 0.08 | | *p* = 0.30 | | *p* = 0.45 | |
| Ipsilateral Lung (*D*_50%_)  (n = 18) | 1.9  [0.5 – 7.8] | 1.7  [0.3 – 6.8] | 1.5  [0.5 – 5.3] | 1.4  [0.5 – 5.3] | 1.6  [0.5 – 5.5] | 1.3  [0.4 – 5.5] |
|  | *p* = 0.14 | | *p* = 0.67 | | *p* = 0.17 | |
| Liver (*D*_700cc_)  (n = 11) | 2.5  [0.6 – 28.1] | 2.4  [0.6 – 25.6] | 2.1  [0.4 – 17.2] | 2.0  [0.4 – 14.7] | 2.8  [0.4 – 18.4] | 2.8  [0.4 – 16.0] |
|  | *p =* 0.10 | | *p =* 0.29 | | *p =* 0.14 | |
